# Harnessing the Metabolic Vulnerabilities of Leukemia Stem Cells to Eradicate Acute Myeloid Leukemia

**DOI:** 10.3389/fonc.2021.632789

**Published:** 2021-03-17

**Authors:** Bijender Kumar

**Affiliations:** Department of Stem Cell Transplantation and Cellular Therapy, MD Anderson Cancer Center, Houston, TX, United States

**Keywords:** leukemia stem cells, metabolism, drug resistance, bone marrow niche, DHODH inhibitor, oxidative phosphorylation, NAMPT

The current opinion is on the Jones et al. (1) study, discovering the nicotinamide metabolic dependency of Venetoclax resistant AML leukemic stem cells, recently published in Cell Stem Cell Nov 2020 issue.

The major therapeutic paradigm for Acute Myeloid Leukemia (AML) treatment has been either allogenic stem cells transplantation or chemotherapy (cytarabine/anthracyclines combinations, 7+3) to induce apoptosis of the proliferating blasts. Conventional chemotherapy in AML often eliminates majority of proliferating leukemic cells, however most of the patients eventually relapse after chemotherapy discontinuation due to the persistence of AML stem cells (LSCs). The conventional wisdom suggests that targeting cancer stem cells (CSCs) results in improved survival rates with chemotherapy combinations by eliminating the possibility of recurrence. John Dick's group had originally identified the AML LSCs in primary patients using serial xenotransplantation experiments in NOD/SCID immunodeficient mice and observed that LSCs reside in lineage-CD34^+^CD38^−^ cells fraction (2, 3). Jordan et al. (4) and many other studies further showed that Interleukin-3 receptor alpha chain (IL-3Rα or CD123) expression in CD34^+^ cells enriches the LSCs frequency (5). The IL-3Rα (IL-3 cytokine receptor) or other LSC markers expression on LSCs confers self-renewal advantage over the normal hematopoietic stem cells (HSCs), provide an increased signaling pathways activation leading to increased blasts counts and clinical drug resistance phenotype. The frequency and potential of LSCs is highly variable in patients and remains the critical determinant for disease evolution and relapse.

AML LSCs and blasts reside in bone marrow (BM) niches, where the nutrients are limited. Hence, the adaptation to the microenvironment and competition for resources are the crucial determinants for successful survival of LSCs and blasts. Given the dismal disease prognosis of relapsed or refractory AML patients, understanding the LSCs specific energy metabolism to impart metabolic programming and identification of their metabolic dependencies is critical to improving outcomes. Jones et al. (1) have recently identified the novel therapeutically targetable mechanism of resistance to Venetoclax in refractory and relapsed AML LSCs, mediated by activation of nicotinamide metabolism rate limiting enzyme, Nicotinamide Phosphoribosyltransferase (NAMPT). Further, NAMPT pharmacological inhibition significantly reduced oxidative phosphorylation (OXPHOS) by lowering the amino acids and fatty acid oxidation (FAO) metabolism into Tricarboxylic acid (TCA) cycle in the relapsed and refractory AML LSCs. NAMPT inhibition thus may be a viable approach to eradicate the Venetoclax resistant AML LSCs, while sparing the normal hematopoiesis (1). Their previous clinical trial data with Venetoclax-Azacytidine combination further suggested that the LSCs in *de-novo* and relapsed/refractory AML are metabolically very distinct and provide a window of opportunity for such poor prognosis patients with NAMPT small molecule inhibitors (APO866 and KPT-9274) along with chemotherapy or Venetoclax based combinations.

Apart from nicotinamide metabolism adaption during disease relapse or refractory stages, earlier studies have shown the leukemic blasts and LSCs have evolved a complex adaptation of metabolic flexibility through cell intrinsic, partly due to the selection pressure from the chemotherapy and through various microenvironmental mechanisms. Gene expression and global metabolomics through Carbon (^13^C), nitrogen (^15^N) isotypes tracing studies show that the various metabolic axes happen simultaneously, through distinct fine-tuning of the balance between all metabolic pathways (Figure 1). The metabolites from glucose or its derivatives, lipids and amino acids fuel the oxidative phosphorylation by directing toward electron transport chain (ETC) for main oxidative form of energy production.

**Figure 1:**
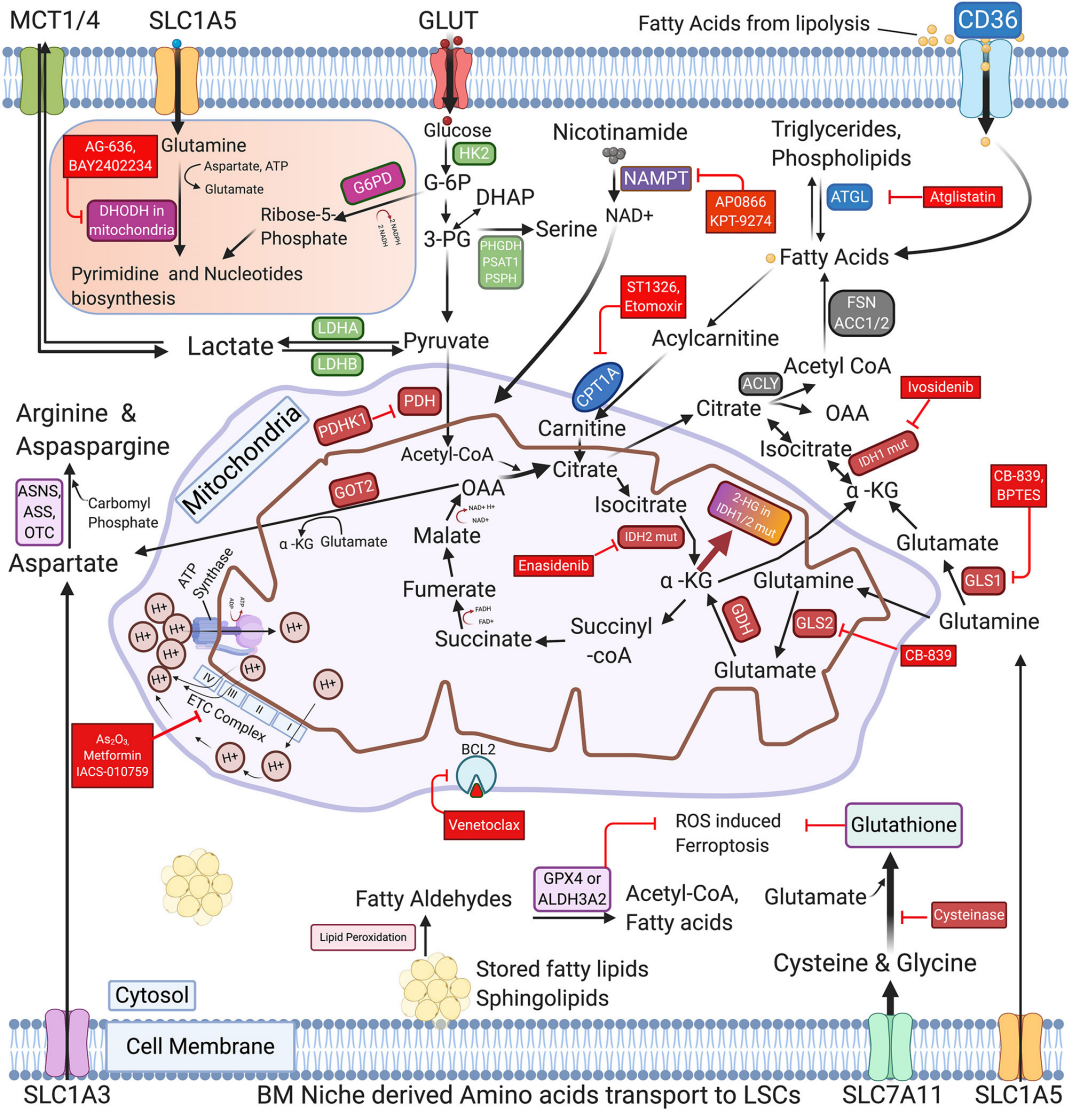
Schematic representation of commonly activated metabolic pathways and therapeutic vulnerabilities of AML blasts and LSCs. The regulation of glucose, Nicotinamide, various amino acids, and Free fatty acids metabolism in AML cells is highly coordinated and interconnected in AML blasts and LSCs. The proliferating AML cells consume glucose using glucose transporters and metabolize it through glycolysis, TCA and OXPHOS to generate ATPs. AML LSCs also express enzymes involved in pyrimidine synthesis, glutaminolysis, nicotinamide metabolism, and fatty acid oxidation (FAO) to generate energy and synthesize nucleic acids. Cysteine and glutamate and other amino acid serve as glutathione precursors to maintain the cellular redox homeostasis by inhibiting the ROS induced Ferroptosis. The figure also depicts the key enzymes that may be promising targets for AML therapy along with their targeting drugs, highlighted in the red boxes. FAO promoting genes are represented in the blue boxes. GLUT, Glucose transporters; MCTs, Lactate transporters; SLC1A5, SLC1A3 & SLC7A11-Amino acid transporters; GLS1/2, Glutaminase-1/2; GDH, Glutamate dehydrogenase; HK2, Hexokinase-II; G6PD, Glucose-6-phospahte dehydrogenase; LDHA/B, Lactate dehydrogenase A/B; IDH1/2 mut, Isocitrate dehydrogenase 1/2 mutations; α-KG, Alpha -ketoglutarate; 2-HG, 2-Hydroxyglutarate; PDH, Pyruvate dehydrogenase; PDHK1, Pyruvate dehydrogenase kinase; DHODH, Dihydroorotate dehydrogenase; PHGDH, Phosphoglycerate dehydrogenase; PSAT1, Phosphoserine aminotransferase; PSPH, Phosphoserine Phosphatase; ACLY, ATP Citrate lyase; ACC1, Acetyl-CoA carboxylase; FASN, Fatty acid synthase; NAMPT, nicotinamide phosphoribosyltransferase; ATGL, Adipose triglyceride lipase; CPT1A, Carnitine Palmitoyltransferase 1A; GOT2, Aspartate aminotransferase; ASS, Argininosuccinate synthase; ASNS, asparagine synthetase; OTC, Ornithine transcarbamylase; ALDH3A2, Aldehyde dehydrogenase 3a2 and GPX4-glutathione peroxidase-4; As_2_O_3_, Arsenic Trioxide; ETC complex, Electron Transport Chain complex (I-IV subunits).

The aberrant overexpression of BCL2 and related anti-apoptotic family genes (BCL-x_L_ and MCL-1) is known to be associated with tumorigenesis and one such adaptation to survive in response to chemotherapy to induce drug resistance in various cancers. Konopleva et al. (6) had identified that BH3 mimetic (ABT-737) effectively killed the AML blasts/LSCs without affecting normal hematopoietic cells. Lagadinou et al. (7) further strengthened the mechanism behind the drug resistance and identified that the majority of functionally defined AML LSCs exhibited significantly reduced levels of ROS and higher BCL2 expression. The successive kinetic studies determined that the BCL2 inhibition induced mitochondrial dysfunctions, leading to energy depletion and eventually leading to eradication of quiescent LSCs. The pioneering work of these studies provided the rationale of targeting the chemotherapy resistant AML LSCs and paved way for the development of pharmacological BCL2 inhibitor, Venetoclax.

AML blasts and LSCs have separate mechanisms to meet the energy demands and quiescence status maintenance. Existing evidence demonstrate that the leukemic blasts and LSCs have significantly higher levels of OXPHOS compared to HSCs and OXPHOS inhibition by Venetoclax is effective in targeting AML cells (8). Further, DiNardo et al. (9) carried out a dose escalation phase-I study in the untreated elderly adults by treating them with Venetoclax and hypomethylating agents (HMA, decitabine, or azacitidine) combinations. The combination was well-tolerated and effective in inducing complete remission in otherwise really poor prognosis patients. We have previously uncovered the possible mechanism of underlying synergistic effect of Venetoclax with HMA in AML and shown that addition of Venetoclax significantly inhibits the AML specific NRF2 gene and its downstream antioxidant pathway activation machinery leading to increased AML blasts LSCs clearance (10).

This study provided proof of principle concept that Venetoclax+ HMA mediated LSC specific amino acid, OXPHOS and antioxidant pathways activation inhibition can serve as curative therapy. Recently, another OXPHOS inhibitor, IACS-010759 clinical trial (NCT02882321) testing is also underway for relapsed and refractory AML. Owing to the high metabolic flexibility the leukemic LSCs in various leukemias, there are few reports of new subclones emerging with higher glycolysis, fatty acid oxidation (FAO), nicotinamide and OXPHOS metabolism resulting in increased resistance to novel drugs affecting the mitochondria, such as Venetoclax and navitoclax.

Apart from glucose driven glycolysis and OXPHOS, another metabolically important aspect is the role of free fatty acids (FFAs), the AML cells switch to alternate energy sources for survival in nutrient deprived conditions due to known vascular dysfunctions in AML. The adipocytes in the hypoxic niches can provide the reservoir of fatty acids for the AML LSCs and blasts. Ye et al. (11) had shown that the gonadal adipose tissue resident CD36^+^ AML LSCs have altered metabolism compared to CD36^−^ LSC and can survive by increasing the FAO driven TCA-OXPHOS to evade the chemotherapy. Similarly, we have previously shown that the AML LSCs alone or through their secreted exosomes create a unique proinflammatory environment that favors leukemia progression at the expense of normal hematopoiesis and skews the MSCs differentiation toward adipocytes lineage, thereby persevering the available energy in the niche for the expanding leukemic blasts instead of spending on the osteoblast differentiation process (12). Additionally, we dissected the leukemia driven BM and adipocytic niches dysfunctions and showed that the AML/ALL LSCs derived exosomes increase adipolysis genes, adipose triglyceride lipase (ATGL) & hormone sensitive lipase (HSL) to increase the FFAs pool for the proliferating leukemic cells to drive their FAO reliant OXPHOS (13).

Since increased proliferation and differentiation blockade of blasts are the hallmarks of AML disease, the approach of differentiation induction in leukemic cells is also of major interest and can effectively cure the disease. The mitochondrial genes, Dihydroorotate dehydrogenase (DHODH) and Isocitrate dehydrogenases (IDH1/2) are overexpressed or mutated in AML and represent important candidates for differentiation therapy. DHODH is essential for rate limiting step for the *de-novo* production of pyrimidines synthesis in the rapidly proliferating blasts. Initial studies have shown that the AML cells may be metabolically dependent and sensitive to pyrimidine starvation compared to normal hematopoietic stem/progenitor cells, thus providing a window of opportunity for therapeutic targeting. Sykes et al. (14) had previously shown that the pharmacological DHODH inhibition leads to reduced AML LSCs survival and the inhibitor can induce their terminal differentiation, indicating that DHODH is a potential differentiation regulator. This has prompted a renewed interest in the clinical development of DHODH inhibitors for clinical translation. Studies have shown that the DHODH inhibitors are highly effective in *in-vitro, in-vivo* pre-clinical leukemia models and currently the clinical efficacy testing of recently developed DHODH inhibitors, BAY2402234 (NCT03404726), and ASLAN003 (NCT03451084) is underway. Apart from DHODH overexpression in AML, the other differentiation induction therapy candidates, Isocitrate dehydrogenase 1/2 (IDH1/2) genes, are frequently mutated (~25%) in AML patients. The IDH gene(s) mutation(s) in AML leads to intracellular oncometabolite 2-hydroxyglutarate (2-HG) accumulation in LSCs/blasts, leading to an altered DNA methylation pattern and increased LSCs persistence due to differentiation blockade, eventually leads to a poor prognosis. The FDA approved IDH inhibitors (Enasidenib and Ivosidenib) have shown very promising results by inducing differentiation and durable remission in the AML patients (15, 16).

Owing to oncogenic signaling and abnormal growth, AML cells are known to have higher levels of oxidative stress from the reactive oxygen species (ROS) compared to non-malignant hematopoietic stem/progenitor cells. Hence, maintenance of the antioxidant genes like aldehyde dehydrogenase (Aldh3a2), Glutathione and its precursor amino acids (Cysteine, glycine and glutamate) are essential for their protection from ferroptosis and blasts proliferation stress induced cell damages (17, 18). Forte et al. (18) and our recent study dissected the mechanism behind the BM niche mediated leukemia survival and showed that despite stroma cells exhaustion with the leukemia progression, the BM derived mesenchymal stem cells (MSCs) continue to thrive (13) and increase OXPHOS in LSCs. The MSCs simultaneously provide the leukemic cells glutathione, necessary to balance ROS levels during leukemogenesis and chemotherapy (18). The leukemic MSCs also reduced the LSCs specific lipid peroxidation damage induced by Cytarabine. Similar findings were reported previously using CLL blasts, showing that the stromal cells promoted glutathione synthesis in CLL cells by transferring cysteine to increase glutathione production and relieving their ROS stress. Further, intracellular Glutathione depletion using pharmacological agent sensitized the CLL to chemotherapy and abrogated the stroma mediated chemoprotection (19). Jones et al. (20) recently showed that the that Cysteine amino acid depletion reduces the ability of AML LSCs to produce Glutathione and eliminates the LSC by inhibition of electron transport complex II energy generation.

Further in- depth investigation on identifying the metabolic dependencies of *de-novo* and relapsed/refractory LSCs will increase our understanding of metabolic vulnerabilities and the survival strategy of leukemia cells. This will facilitate the development of individualized treatments along with Venetoclax/chemotherapy combinations for selective targeting of LSCs to eradicate the disease, while leaving the normal hematopoiesis unperturbed.

## Author Contributions

BK conceived the idea and wrote of the manuscript.

## Conflict of Interest

The author declares that the research was conducted in the absence of any commercial or financial relationships that could be construed as a potential conflict of interest.
